# Mucosal Administration of Recombinant Baculovirus Displaying *Toxoplasma gondii* ROP4 Confers Protection Against *T. gondii* Challenge Infection in Mice

**DOI:** 10.3389/fcimb.2021.735191

**Published:** 2021-09-29

**Authors:** Keon-Woong Yoon, Ki-Back Chu, Hae-Ji Kang, Min-Ju Kim, Gi-Deok Eom, Su-Hwa Lee, Eun-Kyung Moon, Fu-Shi Quan

**Affiliations:** ^1^ Department of Biomedical Science, Graduate School, Kyung Hee University, Seoul, South Korea; ^2^ Department of Medical Zoology, School of Medicine, Kyung Hee University, Seoul, South Korea; ^3^ Medical Research Center for Bioreaction to Reactive Oxygen Species and Biomedical Science Institute, School of Medicine, Graduate School, Kyung Hee University, Seoul, South Korea

**Keywords:** recombinant baculovirus, ROP4, *Toxoplasma gondii* ME49, vaccine, mucosal immunity

## Abstract

Pathogens require physical contact with the mucosal surface of the host organism to initiate infection and as such, vaccines eliciting both mucosal and systemic immune responses would be promising. Studies involving the use of recombinant baculoviruses (rBVs) as mucosal vaccines are severely lacking despite their inherently safe nature, especially against pathogens of global importance such as *Toxoplasma gondii*. Here, we generated rBVs displaying *T. gondii* rhoptry protein 4 (ROP4) and evaluated their protective efficacy in BALB/c mice following immunization *via* intranasal (IN) and oral routes. IN immunization with the ROP4-expressing rBVs elicited higher levels of parasite-specific IgA antibody responses compared to oral immunization. Upon challenge infection with a lethal dose of *T. gondii* ME49, IN immunization elicited significantly higher parasite-specific antibody responses in the mucosal tissues such as intestines, feces, vaginal samples, and brain than oral immunization. Marked increases in IgG and IgA antibody-secreting cell (ASC) responses were observed from intranasally immunized mice. IN immunization elicited significantly enhanced induction of CD4^+^, CD8^+^ T cells, and germinal center B (GC B) cell responses from secondary lymphoid organs while limiting the production of the inflammatory cytokines IFN-γ and IL-6 in the brain, all of which contributed to protecting mice against *T. gondii* lethal challenge infection. Our findings suggest that IN delivery of ROP4 rBVs induced better mucosal and systemic immunity against the lethal *T. gondii* challenge infection compared to oral immunization.

## Introduction

A wide array of replicating and non-replicating viral vectors, such as the adenovirus or the vesicular stomatitis virus have been used for vaccine development against a plethora of diseases. Yet, concerns involving their genotoxicity and potential loss of vaccine efficacy due to pre-existing immunity in recipient hosts resulted in a search for alternative options (Robert-Guroff, 2007). Baculoviruses are a family of DNA viruses with inherently safe aspects, which can address the limitations of other viral vectors. For example, because baculoviruses have a narrow specificity range strictly limited to arthropods, their association with diseases in other species has not been reported (Airenne et al., 2013). In line with this notion, neither cytotoxicity nor pathologies were observed in mammalian cells or animal models following baculovirus infection. Moreover, given that baculoviruses are unable to replicate in mammalian cells, the risk of insertional mutagenesis resulting from genomic integration is non-existent (Kwang et al., 2016). Pairing these intrinsic properties with the absence of pre-existing immunity to baculoviruses in humans (Sung et al., 2014), recombinant baculovirus-based vaccines may be a safe and effective alternative. Resultantly, several baculovirus vaccines displaying the target antigens of various pathogens have been documented, which include the circumsporozoite proteins of *Plasmodium* spp. (Yoshida et al., 2003; Strauss et al., 2007), avian influenza virus (Yang et al., 2007), and others. As exemplified above, incorporating baculoviruses to develop an efficacious vaccine against *Toxoplasma gondii* could bring promising results.

*T. gondii* is an apicomplexan parasite transmitted to humans *via* ingestion of tissue cyst-contaminated food products and is the causative agent of toxoplasmosis (Jones and Dubey, 2012). Currently, approximately a third of the entire world’s population is estimated to be infected with *T. gondii* and their persistence can lead to ocular toxoplasmosis, encephalitis, and even birth defects (Montoya and Liesenfeld, 2004). While the ovine toxoplasmosis vaccine Toxovax is commercially available, the use of this live-attenuated vaccine is strictly prohibited in humans due to safety issues (Dubey, 2009). For this reason, developing an efficacious human toxoplasmosis vaccine is urgent. As of current, only a few baculovirus-based toxoplasmosis vaccine studies have been reported. Gene delivery using pseudotyped recombinant baculovirus (rBV) vaccines expressing *T. gondii* SAG1 and MIC3 antigens conferred better protection against *T. gondii* RH strain than DNA vaccines encoding identical antigens (Fang et al., 2010; Fang et al., 2012). While these studies highlighted the potential use of rBV as a toxoplasmosis vaccine design strategy, neither of the two aforementioned studies investigated the vaccine efficacies against *T. gondii* type II clonal lineage which are more frequently associated with human toxoplasmosis (Arranz-Solís et al., 2019). Moreover, the extent of mucosal immunity induced through baculovirus vaccines against *T. gondii* infection remains unreported to date.

The rhoptry proteins (ROP) of *T. gondii* are key components required for successful parasitic invasion of the host cell (Dubremetz, 2007). ROP4 is one such antigen secreted during the parasitic invasion of host cells, which appears to be associated with the vacuole membrane function (Carey et al., 2004). However, their role extends far beyond host cellular intrusion as these have also been documented to be involved in intracellular parasitic proliferation and virulence (El Hajj et al., 2007). Based on these rationales, vaccines expressing the rhoptry proteins as antigens would be effective at restricting parasitic growth and invasion. Previously, we have generated a virus-like particle (VLP) vaccine expressing the ROP4 antigen of *T. gondii* (Kang et al., 2019). However, high production costs and the requirement for extensive downstream purification processes are the main issues of this vaccine platform (Effio and Hubbuch, 2015; Chu and Quan, 2021). Compared to VLPs, rBVs can be rapidly manufactured in large quantities at cheaper production costs, thus enabling these to be suitable vaccine platforms (Kis et al., 2019). Here, we generated rBV vaccines displaying the rhoptry protein 4 (ROP4) antigen and assessed the mucosal immunity induction, as well as their protective efficacy in mice following lethal challenge infection with the type II *T. gondii* ME49 strain. Our findings revealed that rBV vaccines expressing the ROP4 antigen (ROP4-rBV) were effective inducers of mucosal immunity, as indicated by the robust cellular and humoral responses which contributed to protection against a lethal dose of *T. gondii*.

## Materials and Methods

### Animals and Ethics

Six-week-old female BALB/c mice were purchased from NARA Biotech (Seoul, South Korea). Animals were maintained in approved facilities under specific-pathogen-free conditions with easy access to food and water. All animal experiment protocols have been approved and were conducted following the guidelines of the Kyung Hee University IACUC (permit number: KHUASP (SE) 20-648).

### Parasite, Cells, and Antibodies

Parasites and cells used in the present study were maintained as previously described (Kang et al., 2020b). *T. gondii* ME49 strain was maintained by serial passage in BALB/c mice and ME49 cysts were isolated from the brains. *Spodoptera frugiperda* (Sf9) cells used for rBV production were cultured in spinner flasks with serum-free SF900II media (Invitrogen, Carlsbad, California, USA) at 27°C, 130–135 rpm. Horseradish peroxidase (HRP)-conjugated goat anti-mouse IgG and IgA secondary antibodies were purchased from Southern Biotech (Birmingham, AL, USA).

### Generation of Recombinant Baculovirus

Recombinant baculoviruses were produced following the Bac-to-Bac™ (Thermo Fisher Scientific, Waltham, MA, U.S.) manufacturer’s guidelines. Briefly, ROP4 genes were amplified *via* polymerase chain reaction (PCR) and cloned into pFastBac vector. After subsequent transformation into DH10Bac competent cells, bacmid DNA was acquired and transfected into the Sf9 cells as described (Kang et al., 2019). Single plaque purification was performed in the second passage. To quantify the ROP4-rBVs released into the cell culture supernatants, baculovirus plaque assays were performed using the culture supernatants. Endotoxin presence in the recombinant baculoviruses was checked using Pierce™ Chromogenic Endotoxin Quant Kit (Thermo Fisher Scientific, Waltham, MA, US). To characterize the expression of ROP4 in rBVs, a polyclonal mouse anti-*T. gondii* antibody was used to probe *T. gondii* ROP4 protein in rBVs by ELISA as previously described elsewhere (Welsh, 2011; Lee et al., 2020). Briefly, wells of 96-well plates were coated with serially diluted ROP4 rBVs overnight at 4°C. The wells were incubated with polyclonal mouse anti-*T. gondii* antibody and the secondary HRP-conjugated IgG antibodies. Polyclonal mouse anti-influenza virus antibody was used as a negative control.

### Immunization and Challenge Infection

A total of 24 mice were subdivided into 4 groups (n = 6 per group): unimmunized (naïve), unimmunized mice which were challenge-infected (Naïve+Cha), oral immunization (oral), and intranasal immunization (IN). For prime and boost immunizations through the oral and IN routes, 100 μl of ROP4 rBVs (4.28x10^4^ pfu) were inoculated *via* respective routes at 4 week intervals. Mice were challenge-infected through the oral route with 50 LD_50_ ME49 cysts (2,000 cysts) 4 weeks after boost immunization. All of the mice were monitored daily to record bodyweight changes and survival rates. At 16 days post-infection (dpi), all of the mice were sacrificed for organ sampling and *ex vivo* immunological assay purposes.

### Sample Preparation

Blood samples were collected by retro-orbital plexus puncture 3 weeks after each immunization. Mice were sacrificed at 16 dpi for brain and mucosal sample (intestines, feces, and vaginal secretions) acquisition as previously described (Kang et al., 2019). Briefly, feces were collected and normalized by adding 100 μl of PBS per 0.1 g of feces. Vaginal samples were collected by repeatedly washing the vaginal canal with 200 μl of PBS. Duodenums of mice were longitudinally sliced and immersed in 500 μl of PBS. All of the mucosal samples were processed on an individual basis and incubated for 1 hour at 37°C. Samples were centrifuged at 5000 rpm for 10 min to collect supernatants, which were stored at -20°C until use. Brain tissues were individually processed for cyst burden quantification.

### Antibody Responses

*T. gondii*-specific antibody responses from sera, brain, and mucosal tissues were determined using enzyme-linked immunosorbent assay (ELISA) as previously described (Kang et al., 2019). In brief, flat-bottom 96 well immunoplates (SPL Life Sciences, Pocheon, Korea) were coated with 100 μl of sonicated *T. gondii* ME49 dissolved in carbonate coating buffer (4 μg/ml) overnight at 4°C. Plates were blocked with 0.2% gelatin dissolved in 0.1M PBS with 0.05% Tween 20. Sera and mucosal samples diluted in PBS were used as primary antibodies (1:50 sera, 1:100 intestine, 1:20 vaginal, 1:2 fecal sample dilutions). After incubating the wells with the primary antibodies for 1 hour at 37°C, HRP-conjugated goat anti-mouse IgG and IgA (1:2000 dilution in PBS) secondary antibodies were inoculated into the wells and incubated for 1 hour, 37°C. O-phenylenediamine substrate was dissolved in citrate-phosphate buffer (pH 5.0) containing 0.03% H_2_O_2_ and was used for color development. The optical density at 490 nm was measured using an ELISA reader (EZ Read 400, Biochrom Ltd., Cambridge, UK).

### Antibody-Secreting Cell (ASC) Responses

At 16 dpi, spleens and mesenteric lymph nodes (MLN) were collected from sacrificed mice to assess ASC inductions. Single cell suspensions of splenocytes and MLN cells were prepared as previously described (Kang et al., 2019). Briefly, after RBC lysis, splenocytes and MLN cells were seeded (1 x 10^6^ cells/well) into 96 well plates coated with *T. gondii* ME49 (4 μg/ml). After incubating the cells for 5 days at 37°C, 5% CO_2_, plates were washed and incubated with HRP-conjugated anti-mouse IgG and IgA antibodies for 1 hour, 37°C. Colorimetric assay was performed using OPD substrate and after stopping the reactions with 2N H_2_SO_4_, OD_490_ values were measured.

### Flow Cytometry Analysis of Immune Cell Populations

Splenocytes and MLN cells were prepared for flow cytometric analysis as previously described (Kang et al., 2020a). Single cell suspensions of splenocytes (1 x 10^6^ cells/mouse) and MLN cells (1 x 10^5^ cells/mouse) were stimulated *ex vivo* with *T. gondii* ME49 antigen (2 μg/mL) at 37°C with 5% CO_2_ for 2 hours. After antigen stimulation, cells were stained with the following fluorescent-conjugated antibodies purchased from BD Biosciences (Franklin Lakes, NJ, USA) and Invitrogen (Waltham, MA, USA) for CD4^+^ T cell, CD8^+^ T cell, and germinal center B (GC B) cell detection: CD3 (PE-Cy7), CD4 (FITC), CD8 (PE), GL7 (PE), and B220 (FITC). All staining procedures for flow cytometry were performed according to the manufacturer’s protocol. Stained cells were acquired using the Accuri C6 flow cytometer and analyzed with the C6 Accuri software (BD Biosciences, Franklin Lakes, NJ, USA).

### Inflammatory Cytokine Assays

At 16 dpi, the brain tissues of mice were individually homogenized in 500 μl of PBS. After centrifugation, supernatants were collected for cytokine assay while the pellets were used for brain cyst counting. Pro-inflammatory cytokines IFN-γ and IL-6 were measured from the brain supernatants of *T. gondii*-infected mice using BD OptEIA ELISA kits (BD Biosciences, Franklin Lakes, NJ, USA). All experiments were performed as per manufacturer’s instructions and cytokine concentrations were calculated using the generated standard curve.

### Parasite Burden

*T. gondii* ME49 cysts were isolated from the brains and enumerated as previously described (Kang et al., 2019). After centrifuging the brain homogenates, cysts were isolated using Percoll density gradient cell isolation media (BD Biosciences, Franklin Lakes, NJ, USA). Briefly, sedimented pellets were resuspended in 44% Percoll and gently overlaid on top of 67% Percoll media. After centrifugation at 12,100 rpm for 20 min, the layer containing the *T. gondii* cysts was carefully collected and repeatedly washed with PBS. Cysts were mounted on a clean slide glass and counted under the microscope (Leica DMi8, Leica, Wetzlar, Germany). Cysts were counted from 3 different fields of views per mouse.

### Statistical Analysis

All statistical analyses were compared using the GraphPad Prism version 6 software. (San Diego, CA, USA). Data sets were presented as mean ± SD. Statistical significance between the means of groups was determined using One-way ANOVA with Tukey’s *post hoc* test or 2-way ANOVA with Bonferroni’s *post hoc* test. *P* values (* < 0.05, ** < 0.01, *** < 0.001, **** < 0.0001) were considered statistically significant.

## Results

### Experimental Schedule and Antibody Responses in Immune Sera

**Figure 1:**
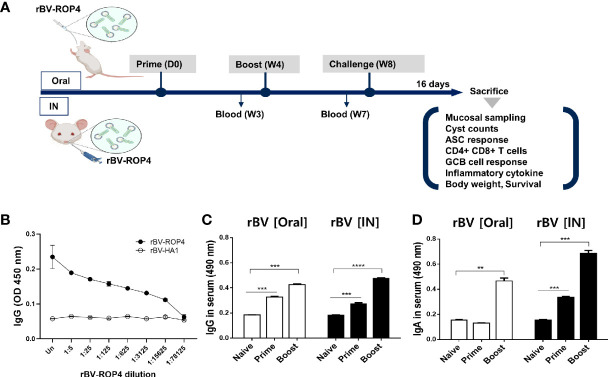
Immunization schematic and *T. gondii* ROP4-specific antibody responses. BALB/c mice were immunized with the ROP4-rBVs vaccine through the oral and intranasal routes with blood collection at regular intervals as scheduled **(A)**. Polyclonal *T. gondii* antibody collected from mice was used to assess ROP4-specificity *via* ELISA **(B)**. Sera were collected 3 weeks after each immunization and *T. gondii* ME49 antigen-specific IgG **(C)** and IgA **(D)** antibody responses were determined by ELISA. Data are presented as mean ± SD and asterisks indicate statistical differences between groups (**P < 0.01, ***P < 0.001, ****P < 0.0001). Images were created with BioRender.com.

The experiment was performed as illustrated in the vaccine prime-boost scheme (**Figure 1A**). ROP4 in rBVs was characterized by ELISA using polyclonal mouse anti-*T. gondii* antibody (**Figure 1B**). Polyclonal *T. gondii* sera successfully reacted with serially diluted ROP4 rBVs while influenza hemagglutinin (HA1) from A/PR/8/34 showed no reactivity with the anti-*T. gondii* antibody, thereby suggesting that the epitope regions of ROP4 protein in the rBVs were similar to that of *T. gondii*. *T. gondii*-specific antibody responses were measured using the sera acquired from mice following prime and boost immunizations. Enhanced IgG response was observed 4 weeks after prime immunization, regardless of vaccine administration route. IgG antibody induction was further elevated 4 weeks after boost immunization (**Figure 1C**). Contrary to the findings observed for IgG, oral immunization of ROP4-rBVs did not lead to enhanced antibody response after prime immunization. However, a noticeable increase was observed after boost immunization. IgA antibody response profile for IN immunization of ROP4-rBVs was similar to IgG, with a drastic increase in antibody induction being observed after boost immunization (**Figure 1D**). When comparing the two immunization groups, IN-immunized mice elicited greater quantities of IgA than orally immunized mice.

### Antibody Responses in Mucosal Samples

**Figure 2:**
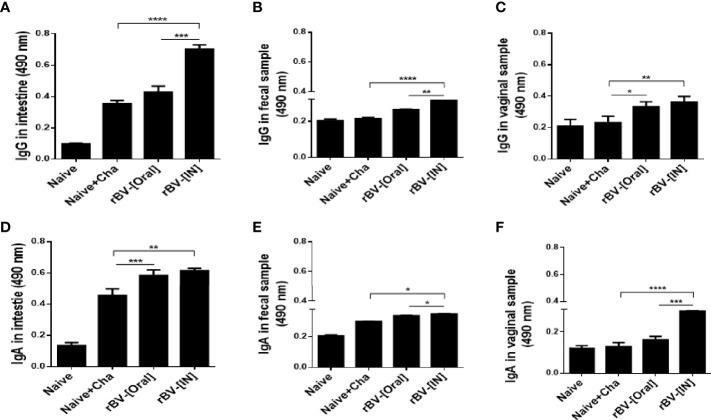
Measurement of enhanced mucosal immune response. *T. gondii*-specific mucosal IgG and IgA responses in the intestines **(A, D)**, feces **(B, E)**, and vaginal samples **(C, F)** were observed using ELISA. Data are presented as mean ± SD and asterisks indicate statistical differences between groups (*P < 0.05, **P < 0.01, ***P < 0.001, ****P < 0.0001).

To assess the extent of mucosal immunity induction, antibody productions in the mucosal tissues of mice were evaluated. At 16 dpi, compared to unimmunized control, increased IgG and IgA responses were observed from the Naïve+Cha group. Immunizing the mice with ROP4-rBVs resulted in greater quantities of *T. gondii*-specific antibody induction, although significant differences between oral immunization and Naïve+Cha were not observed for IgG (**Figures 2A, D**). Compared to Naïve+Cha, significant increases in fecal IgG and IgA were only observed from IN immunization group (**Figures 2B, E**). Immunizing the mice with the ROP4-rBVs resulted in increased IgG responses in the vaginal samples. However, such increase was only observed from IN-immunized mice for the IgA antibody responses (**Figures 2C, F**). Overall, the highest mucosal antibody responses were observed from the intestines.

### Antibody Responses in the Brain

**Figure 3:**
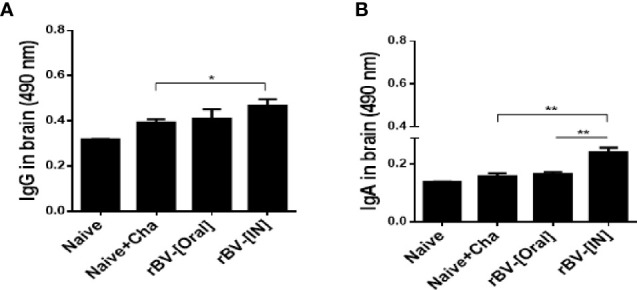
IgG and IgA antibody responses in the brain after *T. gondii* ME49 infection. *T. gondii*-specific IgG **(A)** and IgA **(B)** antibody responses were detected in immunized mice 16 days after challenge infection. Data are presented as mean ± SD and asterisks indicate statistical differences between groups (*P < 0.05, **P < 0.01).

Vaccinating mice with the ROP4-rBVs enabled antibody accumulation in the brains. While the differences in brain IgG responses were comparable for Naïve+Cha and oral immunization group, a marked increase in IgG was observed for IN immunization group (**Figure 3A**). Compared to the naïve control, incremental increases in IgA responses were detected for Naïve+Cha and orally immunized mice. However, IN administration of ROP4-rBVs significantly enhanced the induction of IgA responses in the brain (**Figure 3B**).

### Oral and IN Vaccination Induces Antibody-Secreting Cell Responses

**Figure 4:**
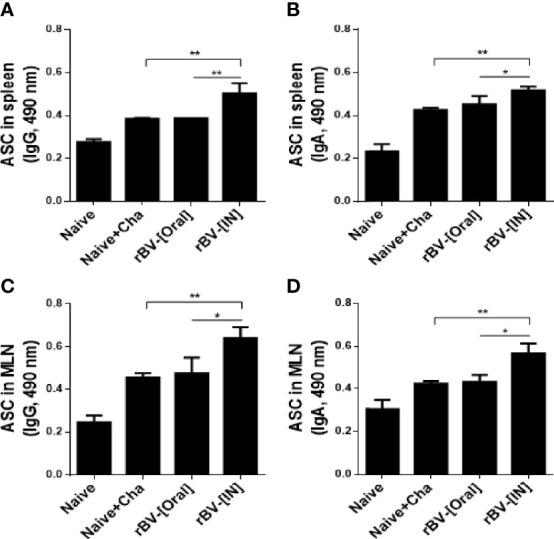
ROP4-rBVs immunization enhances antibody-secreting cell responses. The levels of IgG and IgA antibody-secreting cells were evaluated in spleen and MLN cells. A higher level of *T. gondii*-specific IgG was found in the group of mice immunized with the IN pathway compared to the Naive+Cha group **(A, C)**. The group of mice immunized by the oral route is at a similar level to the Naive+Cha group. The group of mice immunized with the IN route responded significantly to *T. gondii*-specific IgA **(B, D)**. Data are presented as mean ± SD and asterisks indicate statistical differences between groups (*P < 0.05, **P < 0.01).

To determine the antibody-secreting cell response, splenocytes and MLN cells were collected from mice and cultured for 5 days. Differences in IgG and IgA antibody responses were not observed from cultured splenocytes. However, a marked increase in IgG and IgA responses were observed from splenocytes of IN immunization mice (**Figures 4A, B**). Identical findings were also detected from MLN cells as IgG and IgA ASC responses were similar for both Naïve+Cha and oral immunization groups. This was further enhanced by IN immunization with ROP4-rBVs (**Figures 4C, D**).

### Activation of CD4^+^, CD8^+^ T cells, and GC B in MLN and Spleen

**Figure 5:**
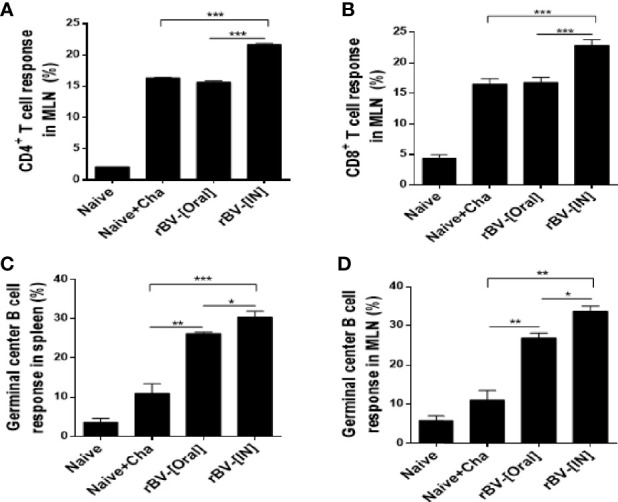
Detection of CD4^+^ T cell, CD8^+^ T cell, and GC B cell. Mice were sacrificed at 16 dpi for spleen and MLN acquisition. Single cell suspensions of splenocytes and MLN cells were prepared and analyzed using flow cytometry to confirm proliferation of CD4^+^ T cell **(A)**, CD8^+^ T cell **(B)**, and GC B cell **(C)** in MLN. Splenic GC B cell activation was also confirmed using flow cytometry **(D)**. Data are presented as mean ± SD and asterisks denote statistical differences between groups. (*P < 0.05, **P < 0.01, ***P < 0.001).

Flow cytometry was performed to assess the proliferation of CD4 and CD8 T cells, as well as GC B cells in the spleens and MLNs of mice. CD4^+^ and CD8^+^ T cell population inductions in the MLN were similar between Naïve+Cha and oral ROP4-rBV immunized mice. However, intranasally administering ROP4-rBVs led to enhanced T cell proliferations (**Figures 5A, B**). GC B cell responses observed from splenocytes were strikingly similar to those of MLN cells. *T. gondii* infection in unimmunized mice elicited marginal GC B cell responses, but this was dramatically elevated upon immunization. Of the two immunization routes, IN route contributed to greater GC B cell response inductions in both spleen and MLN (**Figures 5C, D**).

### Pro-Inflammatory Cytokine Responses in the Brain

**Figure 6:**
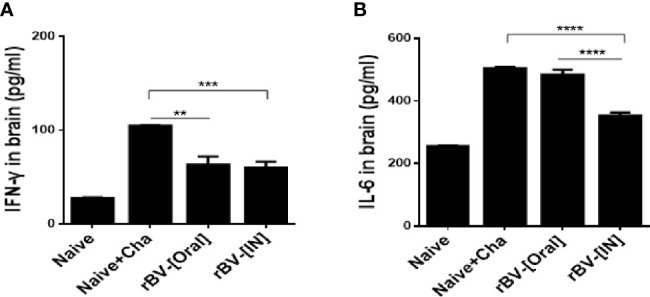
Inhibition of pro-inflammatory cytokines in the brain. Brains of mice were collected 16 days after *T. gondii* challenge infection. The levels of the pro-inflammatory cytokines IFN-γ **(A)** and IL-6 **(B)** were determined in the brain supernatant. Data are presented as mean ± SD and asterisks indicate statistical differences between groups. (**P < 0.01, ***P < 0.001, ****P < 0.0001).

Brain homogenates of mice were used to assess the production of pro-inflammatory cytokines IFN-γ and IL-6. Challenge-infection with *T. gondii* ME49 in unimmunized mice resulted marked rise in IFN-γ production. While such increases were also observed from the brains of immunized mice, IFN-γ was produced to a significantly lesser extent in these groups (**Figure 6A**). IL-6 levels were comparable between Naïve+Cha and oral immunization groups. However, a stark decrease in IL-6 production was detected from IN immunization groups (**Figure 6B**).

### Protective Efficacy of the ROP4-rBV Vaccine

**Figure 7:**
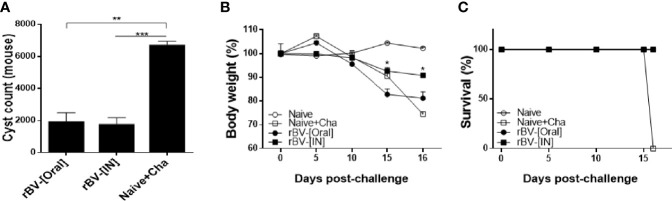
Complete protection against *T. gondii* ME49. Mice were challenge-infected with 50 LD_50_ *T. gondii* ME49 cysts 4 weeks after the final immunization. Mice were sacrificed 16 days post-infection and brain tissues were harvested to isolate and enumerate the cysts **(A)**. Bodyweight changes **(B)** and survival rate **(C)** were monitored daily up to 16 dpi. Data are presented as mean ± SD and asterisks indicate statistical differences between groups. (*P < 0.05, **P < 0.01, ***P < 0.001).

To confirm the protective efficacy of ROP4-rBVs against *T. gondii* ME49 infection, cysts were quantified from the brains of mice under the microscope. More than 6,000 cysts were counted from Naïve+Cha mice, whereas immunized mice underwent a 3-fold reduction in parasite burden (**Figure 7A**). While the cyst counts between the two groups of immunized mice were comparable, bodyweight loss was more apparent in orally immunized mice. At 16 dpi, drastic bodyweight loss exceeding 20% was observed from Naïve+Cha group. Similarly, orally immunized mice also experienced bodyweight reduction approaching the humane intervention point. Yet, IN immunization resulted in 10% bodyweight loss at maximum and retained close to normal bodyweight (**Figure 7B**). Despite the contrasting differences in bodyweight reduction, all of the immunized mice survived (**Figure 7C**). While prolonged survival of immunized mice was highly plausible, all of the mice were sacrificed at 16 dpi to ensure that experiments were carried out on the same day for all groups. These results suggest that the ROP4-rBVs vaccine is more effective when administered *via* IN route.

## Discussion

The intestinal epithelium act as the first line of defense against numerous pathogens including *T. gondii* and mounting a robust mucosal immunity can contribute to limiting the parasitic intrusion of the host cells. While the exact function of ROP4 remains unknown, (Carey et al., 2004) have delineated that ROP4 proteins are heavily involved in vacuole membrane function and subsequently undergo phosphorylation in the infected host cells, which may have further implications. Yet, only a handful of immunization studies using this antigen have been conducted to date. The present study investigated the protective efficacy of a recombinant baculovirus vaccine expressing the *T. gondii* ROP4 antigen. Here, we demonstrated that administering this vaccine through the two mucosal routes induced strong mucosal immune responses that protected mice against a lethal dose of *T. gondii* ME49, with intranasal immunization being better of the two immunization routes.

Oral ingestion of *T. gondii* tissue cysts serves as the natural route of *T. gondii* infection, which readily invades the epithelial cells of the small intestines to initiate infection before disseminating to various organs (Dimier-Poisson et al., 2003; Snyder and Denkers, 2020). A robust mucosal immune response, characterized by antibody responses in the intestines, would act as a barrier and limit *T. gondii* transgressions into the epithelial cells. Baculovirus-based vaccines are capable of inducing mucosal immunity against various mucosal pathogens, as demonstrated using the influenza virus and the human papillomavirus (Fragoso-Saavedra and Vega-López, 2020). Consistent with these findings, the ROP4-rBV vaccine generated in this study successfully induced mucosal antibody responses, especially intestinal IgG and IgA which contributed to protection against a lethal dose of *T. gondii* ME49 infection. This result is supported by the GC B cell proliferation observed from splenocytes as well as the MLN, since the GC B cells act as the site for developing antigen-specific antibody responses (Glatman Zaretsky et al., 2012). Also, it is widely regarded that T cell and B cells are activated following the initial phase of *T. gondii* infection and their expressions regulate parasite replication during the chronic phase of its infection (Glatman Zaretsky et al., 2012). In line with this notion, CD4^+^ and CD8^+^ T cell proliferation were observed from Naïve+Cha mice in our study, which was comparable to those of orally immunized mice. T cell proliferation occurred to a greater extent in the MLN of IN immunized mice than those of orally immunized group or the control groups, implying that IN immunization of ROP4-rBV conferred better protection against chronic toxoplasmosis. These findings can be attributed to the inherent obstacle associated with oral vaccines. Oral vaccines, when administered through the gastrointestinal (GI) tract, are susceptible to denaturation by the proteolytic enzymes that are present in the highly acidic GI tract environment (Vela Ramirez et al., 2017). Exposure to this harsh environmental condition can weaken the antigenicity of the ROP4-rBVs, which may have resulted in weaker mucosal immunity induction than the IN vaccine immunization.

Pro-inflammatory cytokine milieu in the central nervous system is a prominent feature observed during chronic *T. gondii* infection, and persisting neuroinflammations can lead to neurological and psychiatric disorders (Parlog et al., 2015). In general, a pronounced inflammatory cytokine response is one of the features of type II *T. gondii* strains while these are detected to a lesser extent in the clonal lineage types I and III (Xiao et al., 2011; Innes et al., 2019). Our findings revealed that immunizing the mice with the ROP4-rBVs reduced the production of inflammatory cytokines IFN-γ and IL-6 compared to the unimmunized control group. While ROP4-rBV immunization elicited reduced IFN-γ production in the brains irrespective of immunization routes, differences in immunization routes were noticeable for IL-6 with IN immunization inducing less IL-6 production than the oral immunization group.

Though limited in number, the protective efficacies of *T. gondii* vaccines incorporating the ROP4 antigen have been reported. Several studies have investigated the efficacies of subunit cocktail vaccines comprising ROP4 along with various other antigens such as ROP2, GRA4, SAG1, and MAG1 which significantly reduced the cyst burden in mice challenge-infected with *T. gondii* DX strain, a low-virulent type II strain similar to ME49 (Dziadek et al., 2009; Dziadek et al., 2011; Dziadek et al., 2012; Gatkowska et al., 2018). Our previous study also assessed the efficacies of VLP vaccines displaying ROP4 and ROP13 antigens on the surface (Kang et al., 2019). While different strains of *T. gondii* were used for challenge infection, the cyst burden reductions demonstrated by ROP4-rBVs were comparable to the results of the aforementioned multi-antigenic vaccine studies. Immunization-induced proliferation of CD4^+^ T cell, CD8^+^ T cell, and GC B cells in the MLN were consistent with our previous works (Kang et al., 2019).

Noticeable differences in results were observed between the present study and those of our previous studies. Compared to our previous study, discrepancies in mucosal antibody responses were observed which may stem from different vaccine platform usage. The mucosal antibody inductions were much more potent in mice immunized with the ROP4 VLPs than ROP4-rBVs. Notably, while the ROP4-rBVs used in the present study only elicited strong antibody responses in the intestines, ROP4 VLPs from our previous work induced the production of vast quantities of vaginal, urinal, fecal, and intestinal IgG. In many of our previous studies investigating the protective efficacy of *T. gondii* VLP vaccines, unimmunized mice perished around 30 dpi when infected with 450 cysts (Kang et al., 2019; Kang et al., 2020a; Kang et al., 2021a; Kang et al., 2021b). In the present study, unimmunized mice died by 16 dpi which was attributed to the high infection dose exceeding 450 cysts. Nevertheless, cyst burden reductions, bodyweight changes, and survival data of ROP4-rBVs were strikingly similar to those of ROP4 VLPs, thus confirming the efficacy of the rBV vaccines demonstrated here.

In summary, we demonstrated that oral and intranasal immunization with the ROP4-rBV vaccine elicited mucosal immunity which protected mice from lethal challenge infection with the *T. gondii* ME49. In particular, immunological parameters were induced to a greater extent *via* IN immunization. While additional protective efficacy assessment of the ROP4-rBV vaccines against other clonal lineage types is required, the vaccine design strategy presented here may be well-suited for developing a safe and effective *T. gondii* vaccine.

## Data Availability Statement

The original contributions presented in this study are included in the article/supplementary material. Further inquiries can be directed to the corresponding author.

## Ethics Statement

The animal study was reviewed and approved by Kyung Hee University IACUC.

## Author Contributions

F-SQ conceptualized and designed the experiments. K-WY, K-BC, H-JK, M-JK, G-DE, and S-HL performed the experiment and collected the data. K-WY and H-JK analyzed the data. K-WY and K-BC wrote the manuscript. K-BC, E-KM, and F-SQ performed critical revision of the manuscript. All authors contributed to the article and approved the submitted version.

## Funding

This research was funded by the National Research Foundation of Korea (NRF) (2018R1A6A1A03025124) and the Ministry of Health & Welfare, Republic of Korea (HV20C0085, HV20C0142).

## Conflict of Interest

The authors declare that the research was conducted in the absence of any commercial or financial relationships that could be construed as a potential conflict of interest.

## Publisher’s Note

All claims expressed in this article are solely those of the authors and do not necessarily represent those of their affiliated organizations, or those of the publisher, the editors and the reviewers. Any product that may be evaluated in this article, or claim that may be made by its manufacturer, is not guaranteed or endorsed by the publisher.
